# 192. Evaluation Of Oral Vancomycin for Primary Clostridioides difficile Prophylaxis in Hematopoietic Stem Cell Transplant Recipient Patients

**DOI:** 10.1093/ofid/ofaf695.067

**Published:** 2026-01-11

**Authors:** Marina Samuel, Frank Cirrone, Rachel Abramova, Yanina Dubrovskaya, John Papadopoulos, Sarah E Hochman, Kassandra Marsh

**Affiliations:** NYU Langone Health, New York, New York; AstraZeneca, New York, New York; NYU Langone Health, New York, New York; NYU Langone Health, New York, New York; NYU Langone Health, New York, New York; NYU Langone Health, New York, New York; NYU Langone Health, New York, New York

## Abstract

**Background:**

The risk of *Clostridioides difficile* infection (CDI) is high in patients undergoing hematopoietic stem cell transplant (HSCT) due to many factors including prolonged use of antibiotics. Our institution implemented a protocol incorporating primary oral vancomycin prophylaxis (OVP) during the index HSCT admission in June 2021.

**Figure:**
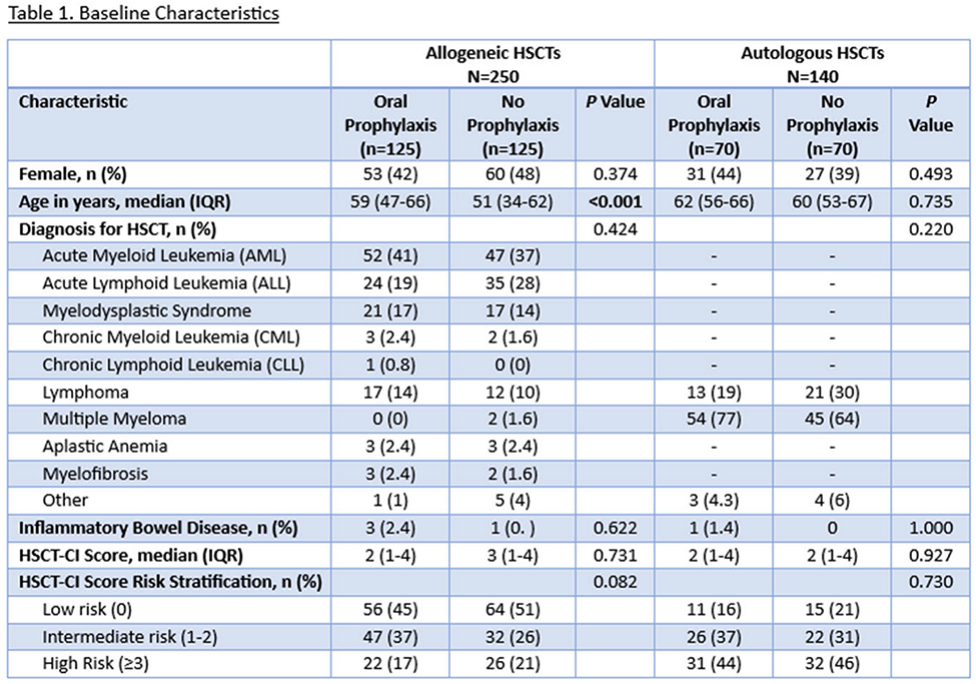

**Figure:**
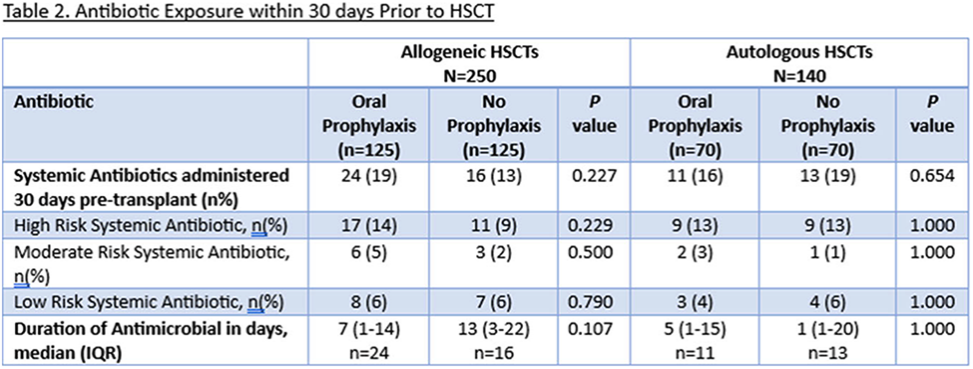

**Methods:**

This is a retrospective review of adult patients who underwent allogeneic (allo) or autologous (auto) HSCT between 2016-2024. Patients in alloHSCT and autoHSCT cohorts were assessed in groups based on pre- compared to post-implementation of the OVP protocol (pre vs post-June 2021; no OVP vs OVP). In the OVP groups, HSCT patients received OVP 125 mg every 12 hours at initiation of bacterial prophylaxis and continued until day of discharge. The primary outcome was CDI incidence during index HSCT admission, defined as a positive CDI test plus receipt of treatment dose vancomycin or fidaxomicin. Secondary outcomes included incidence of CDI within 30- and 60-days post-HSCT, length of stay, vancomycin-resistant *Enterococcus* (VRE) infection, culture-confirmed infection, and gastrointestinal graft versus host disease (GI-GVHD) within 120 days.

**Figure:**
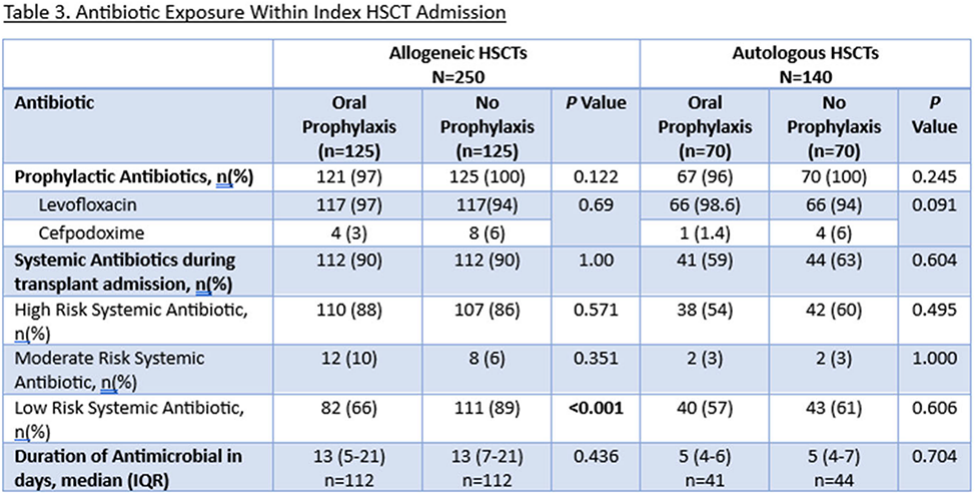

**Figure:**
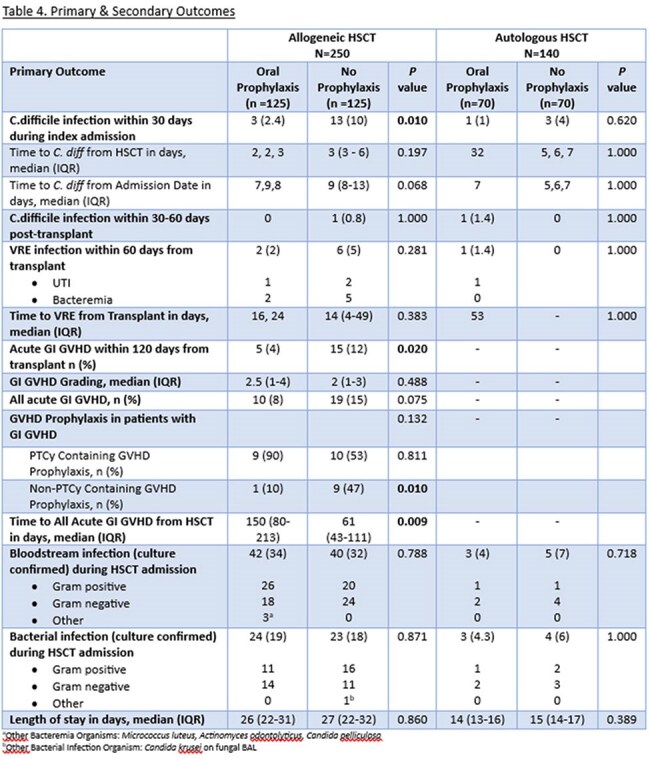

**Results:**

After screening 425 patients, we included 390 (Allo n=250 [125 OVP vs 125 no OVP], Auto n=140 [70 OVP vs 70 no OVP]). Baseline characteristics including sex, hematologic diagnosis, and HSCT-CI score were similar between groups for both cohorts. Among alloHSCT, more patients receiving OVP had peripheral blood graft source (97% vs. 88%, *p*=0.009) and post-transplant cyclophosphamide-based GVHD prophylaxis (97% vs. 73%, *p*< 0.001). Systemic antibiotic use was similar between groups in both cohorts. Median duration of OVP was 23 days (IQR 17-29) and 15 days (IQR 9-17) in the allo and autoHSCT cohorts, respectively. There was a reduced incidence of CDI with use of OVP (Allo 2.4% vs 10%, p=0.010, Auto 1% vs 4% p=0.620). There was no difference in rates of VRE or length of stay in either cohort, but among alloHSCT there was less 120-day GI-GVHD in the OVP group (4% vs 12%, p=0.02).

**Conclusion:**

Among AlloHSCT, there was a reduced incidence of CDI without an increase in VRE infection with the use of primary OVP prophylaxis. We found a similar trend among AutoHSCT patients, though larger studies are required to confirm these findings.

**Disclosures:**

All Authors: No reported disclosures

